# Methionine Supplementation Affects Metabolism and Reduces Tumor Aggressiveness in Liver Cancer Cells

**DOI:** 10.3390/cells9112491

**Published:** 2020-11-16

**Authors:** Farida Tripodi, Beatrice Badone, Marta Vescovi, Riccardo Milanesi, Simona Nonnis, Elisa Maffioli, Marcella Bonanomi, Daniela Gaglio, Gabriella Tedeschi, Paola Coccetti

**Affiliations:** 1 Department of Biotechnology and Biosciences, University of Milano-Bicocca, 20126 Milan, Italy; beatrice.badone@unimib.it (B.B.); m.vescovi2@campus.unimib.it (M.V.); r.milanesi2@campus.unimib.it (R.M.); marcella.bonanomi@unimib.it (M.B.); 2 DIMEVET–Department of Veterinary Medicine, University of Milano, 20133 Milan, Italy; simona.nonnis@unimi.it (S.N.); elisa.maffioli@unimi.it (E.M.); gabriella.tedeschi@unimi.it (G.T.); 3 CRC “Innovation for Well-Being and Environment (IWE)”, University of Milano, 20133 Milan, Italy; 4 SYSBIO.ISBE.IT, Centre of Systems Biology, 20126 Milan, Italy; daniela.gaglio@ibfm.cnr.it; 5 Institute of Molecular Bioimaging and Physiology (IBFM), National Research Council (CNR), Segrate, 20090 Milan, Italy

**Keywords:** AMPK, TCA cycle, migration, growth, proteomics, metabolomics, HCC

## Abstract

Liver cancer is one of the most common cancer worldwide with a high mortality. Methionine is an essential amino acid required for normal development and cell growth, is mainly metabolized in the liver, and its role as an anti-cancer supplement is still controversial. Here, we evaluate the effects of methionine supplementation in liver cancer cells. An integrative proteomic and metabolomic analysis indicates a rewiring of the central carbon metabolism, with an upregulation of the tricarboxylic acid (TCA) cycle and mitochondrial adenosine triphosphate (ATP) production in the presence of high methionine and AMP-activated protein kinase (AMPK) inhibition. Methionine supplementation also reduces growth rate in liver cancer cells and induces the activation of both the AMPK and mTOR pathways. Interestingly, in high methionine concentration, inhibition of AMPK strongly impairs cell growth, cell migration, and colony formation, indicating the main role of AMPK in the control of liver cancer phenotypes. Therefore, regulation of methionine in the diet combined with AMPK inhibition could reduce liver cancer progression.

## 1. Introduction

Liver cancer is the second leading cause of cancer-related death worldwide. Hepatocellular carcinoma (HCC) is the most common form of primary liver cancer and is associated with chronic liver damage, which can be caused by viral infections, such as hepatitis B virus (HBV) and hepatitis C virus (HCV) infection or by alcoholic liver diseases and non-alcoholic steatohepatitis (NASH). Potentially curative treatments for very early/early stage HCC patients include surgical resection, liver transplantation and percutaneous ablation. At an advanced/late stage surgery is no longer applicable, and the currently available therapies are effective only in small groups of patients [1,2,3]. Very few therapeutic options, with unsatisfactory antitumor effects and toxicity, are nowadays available, thus, prognosis remains very poor. In 2007 Sorafenib was the first VEGFR TKI (vascular endothelial growth factor receptor (VEGFR)-tyrosine kinase inhibitor (TKI)) to be approved in advanced HCC, and it is used as standard treatment for patients who have no/mild cirrhosis. However, it gives a significant, but moderate improvement in median overall survival [4]. More recently, other drugs against HCC have been approved, such as Regorafenib, Lenvatinib, and Cabozantinib [4].

AMP-activated protein kinase (AMPK) is a heterotrimeric Ser/Thr protein kinase, highly conserved from yeast to humans. It functions as a sensor of the cellular energy status: in response to metabolic stress AMPK is activated by phosphorylation on a conserved threonine residue (T172), resulting in the upregulation of adenosine triphosphate (ATP)-producing pathways (catabolism) and in the downregulation of ATP-consuming pathways (anabolism), in order to restore energy homeostasis [5]. Downregulation of AMPK in several tumor tissues has been associated with the loss of control in tumorigenesis, cell cycle progression, proliferation and survival, invasion and metastasis, cancer metabolism, and drug resistance. However, its role in carcinogenesis is still controversial, being now considered a double-edged sword that can have either pro-tumor or anti-tumor functions depending on cellular and metabolic demand [6]. The catalytic AMPK-α2 subunit is frequently under-expressed in human HCC cells and inactivation of AMPK promotes hepato-carcinogenesis [7]. It was also found that patients with cirrhosis that present low AMPK phosphorylation had a significant higher incidence of HCC than patients with high phospho-AMPK levels [8]. In addition, low phospho-AMPK staining is correlated with aggressive clinicopathologic features and poor prognosis in patients with HCC [9]. For these reasons, the activation of AMPK in patients with liver cancer has been proposed as a clinical target. Metformin, the widely used anti-diabetics drug, is the most commonly used activator of AMPK, with well-documented pharmacokinetic and safety profiles [10]. However, clinical evaluation of metformin effectiveness alone, or in combination with Sorafenib, has given no clear results yet [11].

Liver is the organ where 50% of methionine metabolism and where 85% of all transmethylation reactions take place [12]. Methionine is an essential amino-acid, particularly abundant in animal food and seafood, but also in nuts, seeds, and cereals, while it is less abundant in fruits and vegetables [13]. In the liver, methionine is converted into S-adenosyl-methionine (SAM), an important cofactor for transmethylation reactions [14]. SAM is also involved in the trans-sulfuration pathway, particularly active in the liver to give cysteine biosynthesis, the rate-limiting precursor for glutathione (GSH) synthesis. SAM is also required for the synthesis of polyamines, small positively charged molecules involved in transcription, translation, cell growth, and death [15].

Methionine restriction is associated with the increase of lifespan in different models (yeast, *Drosophila*, human fibroblasts, rodents [13]) and to the inhibition of cell growth in different cancer cells, such as sarcoma, melanoma, prostate carcinoma, colorectal cancer cells in vitro, as well as in animal models [16].

In general, a different regulation should be assumed in liver cancer cells, considering the peculiar role of the liver in both the synthesis and in the degradation of methionine. SAM is synthesized by methionine adenosyl-transferase (MAT), encoded by two genes (MAT1A and MAT2A). MAT1A is expressed in adult differentiated liver, while MAT2A is expressed in extrahepatic tissues and fetal liver. Liver carcinogenesis is associated to a switch between MAT1A and MAT2A, with a consequent less efficient methionine metabolism, that enhances the capacity of hepatocytes and hepatoma cells to utilize nutrients for anabolic processes to support growth [17]. In addition, MAT1A and GNMT (glycine N-methyltransferase) knockout mice, with very low and very high SAM levels, respectively, spontaneously develop HCC, further supporting the crucial role of methionine-cycle homeostasis for this organ [12,18].

Strikingly, it was recently published that SAM supplementation inhibits liver cancer cell invasion in vitro [19] and decreases liver cancer formation in a mouse model [20]. Additionally, a quite old clinical trial on patients with alcoholic cirrhosis and less severe hepatic dysfunction demonstrated the beneficial role of SAM treatment, by increasing GSH levels and decreasing the probability of liver transplantation and death [21].

Thus, since methionine/SAM administration in liver seems to have an opposite role than in other cancer types, more studies are necessary to support a therapeutic role of methionine or SAM supplementation in patients.

Here, we investigate the effect of methionine supplementation in liver cancer cells in vitro, to explore the possibility that alterations in methionine dietary consumption could help treatment of liver cancer.

## 2. Materials and Methods

### 2.1. Cell Cultures

HepG2 liver cancer cells [22], SW480 colorectal cancer cells [23], A549 lung cancer cells [24], and MCF7 breast cancer cells [25] were obtained from ATCC. Huh7 liver cancer cells were provided by the Japanese Collection of Research Bioresources (JCRB) Cell Bank [26].

HepG2, Huh7, SW480 and A549 cells were cultured using RPMI1640 supplemented with 10% (*v*/*v*) FBS, 2 mM L-glutamine, 100 U/mL penicillin, and 100 μg/mL streptomycin. MCF7 were cultured using EMEM/NEAA supplemented with 10% (*v*/*v*) FBS, 2 mM L-glutamine, 100 U/mL penicillin, and 100 μg/mL streptomycin. Cells were maintained at 37 °C in a humidified 5% CO_2_ incubator. Methionine was dissolved in water at 45 mg/mL and added at a final concentration of 1.5 mg/mL to RPMI1640 medium; in the control medium, the same amount of water was added. Compound C (Calbiochem, San Diego, CA, USA) was dissolved in DMSO and added to the medium at a final concentration of 2 μM for HepG2, SW480, A549, and MCF7 cells, and at 1.5 μM for Huh7 cells.

### 2.2. Growth Curves

1.5 × 10^5^ cells were plated in 6 well plates, the day after the medium was changed (control medium, high methionine, Compound C or High methionine and Compound C). Cells were counted at *t* = 0, 48 h and 72 h.

### 2.3. Migration Assay

Cell migration was assessed using transwell permeable supports (Costar) with 8.0 μm filter membranes. Cells were treated with high methionine and/or Compound C for 24 h, and then serum starved for 24 h. 5 × 10^4^ HepG2 cells and 3.5 × 10^4^ Huh7 cells were resuspended in 100 μL of serum free medium (always in the presence or absence of high methionine and/or Compound C), plated onto each filter and 500 μL of complete medium (containing 10% FBS) were placed in the lower chamber. After 24 h, filters were washed, fixed and stained with 0.5% Coomassie brilliant blue (in 10% acetic acid, 45% methanol). Cells on the upper surface of the filters were removed with cotton swabs. Cells that had invaded to the lower surface of the filter were counted under the microscope.

### 2.4. Clonogenic Assay

A total of 2500 cells were plated in a 6 well plates, treated with high methionine and/or Compound C for 10–15 days (the medium was changed every 3–4 days). Then, colonies were fixed with 70% ethanol for 5 min, stained with 0.5% crystal violet in 10% ethanol for 15 min, finally, washed with water and manually counted.

### 2.5. Total Protein Extraction and Western Blot

Total cell extracts were prepared using RIPA buffer (50 mM Tris-HCl pH 7.5, 150 mM NaCl, 0.5% sodium deoxycholate, 1% NP40, 0.1% SDS), plus 1 mM PMSF (phenylmethanesulfonylfluoride), protease inhibitor cocktail (Roche, Indianapolis, IN) and phosphatase inhibitor cocktail (Sigma-Aldrich, St. Louis, MO, USA). Protein concentration was determined using the Bio-Rad protein assay. Western blot analysis was performed using anti-AMPK antibody (Cell Signaling), anti-phosphoT172-AMPK antibody (Cell Signaling), anti-vinculin antibody (Sigma-Aldrich), anti-phospho-T389-p70 S6K (Cell Signaling, kindly provided by Evelina Gatti), anti-phospho79-Acc1 antibody (Cell Signaling), anti-Akt (Cell Signaling) anti-phosphoS473-Akt (Cell Signaling), anti-tubulin (Cell Signaling).

### 2.6. Small-Interfering RNA-Mediated Gene Silencing

To silence AMPK α/α’, we used RNA interference by using small-interfering RNA (siRNA). Reverse transfection was performed on HepG2 and Huh7 cells with control siRNA (control siRNA-C, Santa Cruz Biotechnology) or siAMPKα/α’ (Santa Cruz Biotechnology, Heidelberg, Germany) specific oligos by using the Lipofectamine 2000 reagent (Invitrogen, Carlsbad, CA, USA). AMPKα/α’ expression was detected by immunoblotting to confirm the silencing achievement.

### 2.7. Shotgun Mass Spectrometry and Label Free Quantification

Four technical replicates were performed for each HepG2 sample, grown for 48 h in the presence or absence of high methionine and/or Compound C. Proteins were lysed in RapiGest 0.1% (RG, Waters Corporation, Milford, MA, USA), reduced with 13 mM DTE (30 min at 55 °C) and alkylated with 26 mM iodoacetamide (30 min at 23 °C). Protein digestion was performed using sequence-grade trypsin (Roche) for 16 h at 37 °C using a protein/trypsin ratio of 20:1. The proteolytic digested was desalted using Zip-Tip C18 (Millipore, Burlington, MA, USA) before MS analysis [27]. LC-ESI-MS/MS analysis was performed on a Dionex UltiMate 3000 HPLC System with a PicoFrit ProteoPrep C18 column (200 mm, internal diameter of 75 μm). Gradient: 2% ACN in 0.1% formic acid for 10 min, 2–4% ACN in 0.1% formic acid for 6 min, 4–30% ACN in 0.1% formic acid for 147 min, and 30–50% ACN in 0.1% formic for 3 min, at a flow rate of 0.3 μL/min. The eluate was electrosprayed into an LTQ OrbitrapVelos (Thermo Fisher Scientific, Bremen, Germany) through a Proxeon nanoelectrospray ion source (Thermo Fisher Scientific), as reported in [28]. The LTQ-Orbitrap was operated in positive mode in data-dependent acquisition mode to automatically alternate between a full scan (*m*/*z* 350–2000) in the Orbitrap (at resolution 60,000, AGC target 1,000,000) and subsequent CID MS/MS in the linear ion trap of the 20 most intense peaks from full scan (normalized collision energy of 35%). Data acquisition was controlled by Xcalibur 2.0 and Tune 2.4 software (Thermo Fisher Scientific).

A database search was conducted against the Homo Sapiens Uniprot sequence database (release 6 March 2019) with MaxQuant (version 1.6.0.1) software, using the following parameters: the initial maximum allowed mass deviation of 15 ppm for monoisotopic precursor ions and 0.5 Da for MS/MS peaks, trypsin enzyme specificity, a maximum of 2 missed cleavages, carbamidomethyl cysteine as fixed modification, N-terminal acetylation, methionine oxidation, asparagine/glutamine deamidation and serine/threonine/tyrosine phosphorylation as variable modifications. Quantification was performed using the built in XIC-based label-free quantification (LFQ) algorithm using fast LFQ [29]. False protein identifications (1%) were estimated by searching MS/MS spectra against the corresponding reversed-sequence (decoy) database. Statistical analysis was performed using the Perseus software (version 1.5.5.3, https://maxquant.net/perseus/). Only proteins present and quantified in at least 75% of the repeats were positively identified and used for statistical analysis. An ANOVA test (cut-off at 0.05 *p*-value) was carried out to identify proteins differentially expressed among the four conditions. Focusing on specific comparisons, proteins were considered differentially expressed if they were present only in one condition or showed significant *t*-test difference (Student’s *t*-test *p* value ≤ 0.05) [30]. Bioinformatic analyses were carried out by Ingenuity^®^ Pathway Analysis software (IPA^®^ - QIAGEN) to cluster enriched annotation groups of Biological Processes, Pathways, and Networks within the set of identified proteins. Functional grouping was based on Fischer’s exact test *p* value ≤ 0.05 (i.e., −log10 ≥ 1.3) and at least 3 counts. Comparison between the proteomic and metabolomic data was performed by IPA and by MetaboAnalyst software R3.0 [31]. Enrichment analysis aimed to evaluate whether the observed genes and metabolites in a particular pathway are significantly enriched (Fisher’s exact test 0.05), while the topology analysis aimed to evaluate whether a given gene or metabolite plays an important role in a biological response, based on its position within a pathway (pathway impact).

The mass spectrometry proteomics data have been deposited to the ProteomeXchange Consortium via the PRIDE partner repository, with the dataset identifier 7MlKNZqC.

### 2.8. Chemicals for Metabolomics Analysis

All chemicals and solvents used for extraction buffer and for liquid chromatography were LC-MS Chromasolv purity grade. Acetonitrile, methanol, 2-Propanol, and water was purchased from Honeywell, while chloroform and formic acid were purchased from Sigma-Aldrich.

### 2.9. Metabolites Extraction for GC-MS Analysis

Cells were quickly rinsed with 0.9% NaCl and quenched with 800 µL of 1:1 ice-cold methanol:water and collected by scraping. Cells were sonicated 5 s for 5 pulses at 70% power twice and then 400 µL of chloroform were added. Samples were vortexed at 4 °C for 20 min, and then centrifuged at 12,000 g for 10 min at 4 °C. The aqueous phase was collected in a glass insert for solvent evaporation in a centrifugal vacuum concentrator (Concentrator plus/ Vacufuge^®^ plus, Eppendorf) at 30 °C for about 2.5 h.

### 2.10. Metabolites Extraction for LC-MS Analysis

Cells were quickly rinsed with 0.9% NaCl and quenched with 500 µL ice-cold 70:30 acetonitrile-water. Plates were placed at −80 °C for 10 min, then the cells were collected by scraping. Cells were sonicated as above and then centrifuged at 12,000 g for 10 min at 4 °C. The supernatant was collected in a glass insert and dried as above. Samples were then resuspended with 150 μL of H_2_O prior to analyses.

### 2.11. GC-MS Metabolic Profiling

Derivatization was performed using automated sample prep WorkBench instrument (Agilent Technologies, Santa Clara, CA, USA). Dried polar metabolites were dissolved in 60 μL of 2% methoxyamine hydrochloride in pyridine (ThermoFisher) and held at 40 °C for 6 h. After the reaction, 90 μL of MSTFA (N-Methyl-N-(trimethylsilyl) trifluoroacetamid) was added, and samples were incubated at 60 °C for 1 h. Derivatized samples were analyzed by GC-MS using a DB-35MS column (30 m × 0.25 mm i.d. × 0.25 µm) installed in an Agilent 7890B gas chromatograph (GC) interfaced with an Agilent 7200 Accurate-Mass Quadrupole Time-of-Flight (QTOF) mass spectrometer (MS), operating under electron impact (EI) ionization at 70 eV. Samples (1 μL) were injected in a splitless mode at 250 °C, using helium as the carrier gas at a flow rate of 1 mL/min. The GC oven temperature was held at 100 °C for 2 min and increased to 325 °C at 10 °C/min. GC/MS data processing was performed using Agilent Muss Hunter software. Relative metabolites abundance was carried out after normalization to internal standard d27 Myristic acid and cell number and statistical analyses were performed using MetaboAnalyst 4.0 [32].

### 2.12. LC-MS Metabolic Profiling

LC separation was performed using an Agilent 1290 Infinity UHPLC system and an InfinityLab Poroshell 120 PFP column (2.1 × 100 mm, 2.7 μm; Agilent Technologies). Mobile phase A was water with 0.1% formic acid. Mobile phase B was acetonitrile with 0.1% formic acid. The injection volume was 15 μL and LC gradient conditions were: 0 min: 100% A; 2 min: 100% A; 4 min: 99% A; 10 min: 98% A;11 min: 70% A; 15 min: 70% A; 16 min: 100% A with 5 min of post-run. Flow rate was 0.2 mL/min, and the column temperature was 35 °C. MS detection was performed using an Agilent 6550 iFunnel Q-TOF mass spectrometer with Dual JetStream source operating in negative ionization mode. MS parameters were: gas temp: 285 °C; gas flow: 14 l/min; nebulizer pressure: 45psig; sheath gas temp: 330 °C; sheath gas flow: 12 l/min; VCap: 3700 V; Fragmentor: 175 V; Skimmer: 65 V; Octopole RF: 750 V. Active reference mass correction was through a second nebulizer using masses with *m*/*z*: 112.9855 and 1033.9881 dissolved in the mobile phase 2-propanol-acetonitrile-water (70:20:10 *v*/*v*). Data were acquired from *m*/*z* 60–1050. Data analysis and isotopic natural abundance correction was performed with MassHunter ProFinder (Agilent Technologies). Relative metabolites abundance was carried out after normalization to cell number and statistical analyses were performed using MetaboAnalyst 4.0 [32].

### 2.13. Metabolites Quantification in the Media Samples

Absolute quantification of glucose, lactate, glutamine, and glutamate in spent media was determined enzymatically using YSI2950 bioanalyzer (YSI Incorporated, Yellow Springs, OH, USA).

### 2.14. Bioenergetics by Seahorse Technology

Mitochondrial oxygen consumption rate (OCR) and extracellular acidification rate (ECAR) were determined by using Seahorse XFe96 Analyzer (Agilent Technologies). HepG2 and Huh7 cells were seeded in Agilent Seahorse cell culture microplates at density of 2 × 10^4^ cells/well for HepG2 or 1 × 10^4^ cell/well for Huh7 72 h prior to the assay. A total of 24 h after seeding cells were treated with high methionine (1.5 g/L) and/or Compound C (2 μM for HepG2 and at 1.5 μM for Huh7 cells) for 48 h, and then analyzed by using the Seahorse XF ATP rate assay kit (Agilent Technologies), according to manufacturer instructions. Three measurements of OCR and ECAR were taken for the baseline and after the sequential injection of mitochondrial inhibitors (1.5 µM oligomycin and 1.5 µM rotenone/antimycin A). OCR and ECAR from each well were normalized by protein content by using the Bradford assay.

## 3. Results

### 3.1. High Methionine and Compound C Induce Proteomic Changes

We recently published findings that methionine activates AMPK and increases mitochondrial metabolism and respiration in the model organism *Saccharomyces cerevisiae*, especially in cells lacking Snf1/AMPK activity [33].

**Figure 1:**
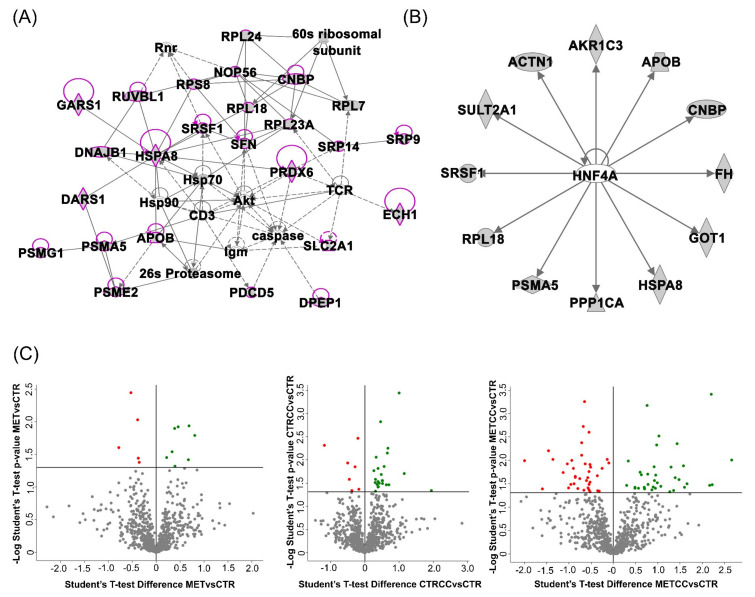
Bioinformatic analysis of the proteomic data of HepG2 cells grown for 48 h (CTR), in the presence of Compound C (CTRCC), high methionine (MET) and high methionine and Compound C (METCC). (**A**) Ingenuity^®^ Pathway Analysis (IPA) analysis carried out on the 46 proteins common to all data sets, whose expression is statistically different, highlighted that 25 out of 46 proteins differentially expressed belong to a network classified as: cancer, protein synthesis, RNA damage and repair. These proteins are shown in magenta among all the proteins belonging to the pathway. Each protein is reported with the corresponding IPA symbol. (**B**) The upstream regulator analysis, based on prior knowledge of expected effects between transcriptional regulators and their target genes in IPA, shows hepatocyte nuclear factor 4 (HNF4A) (*p*-value 0.001) which target 12/46 of the proteins differentially expressed. (**C**) Volcano plots of the comparison MET versus CTR, METCC versus MET, CTRCC versus CTR. In green: proteins upregulated; in red: proteins downregulated; in grey: proteins that are not statistically different (Student’s *t*-test *p*-value = 0.05).

To investigate whether this is a conserved mechanism triggered by methionine, we explored the effect of high methionine concentrations in liver cancer cells, in the presence or absence of Compound C, which mimics AMPK inhibition. We performed a proteomic analysis on HepG2 cells grown for 48 h (CTR), in the presence of Compound C (CTRCC), in the presence of high methionine (MET), and in double treated cells (METCC) by a quantitative shotgun label free strategy. The comparison of the four data sets by an ANOVA test (*p*-value 0.05) showed proteins exclusively expressed in each condition, as well as 717 proteins common to all data sets, among which, 46 were statistically different. IPA analysis carried out on these proteins to find possible interactions highlighted that 25 out of 46 proteins differentially expressed belonged to a network classified as: cancer, protein synthesis, RNA damage and repair (Figure 1A). The upstream regulator analysis, based on prior knowledge of expected effects between transcriptional regulators and their target genes in IPA, suggested that two transcription regulators were probably involved, hepatocyte nuclear factor 1 (HNF1A, *p*-value 0.0005) and hepatocyte nuclear factor 4 (HNF4A, *p*-value 0.001) (Figure 1B), which target 6/46 and 12/46 of the proteins differentially expressed, respectively. This result suggests that the presence of methionine and Compound C significantly affects transcription, in keeping with Wang and coworkers who reported an impact on methionine metabolism by HNF4A [34].

Specific analyses were then carried out by comparing MET versus CTR, METCC versus MET, CTRCC versus CTR. A summary of the results obtained is reported in Figure 3, while Table S2, Table S3 and Table S4 report the list of the proteins differentially expressed (either increased or decreased), or exclusively expressed in one condition in the three comparisons. The corresponding volcano plots are shown in Figure 1C.

**Figure 2:**
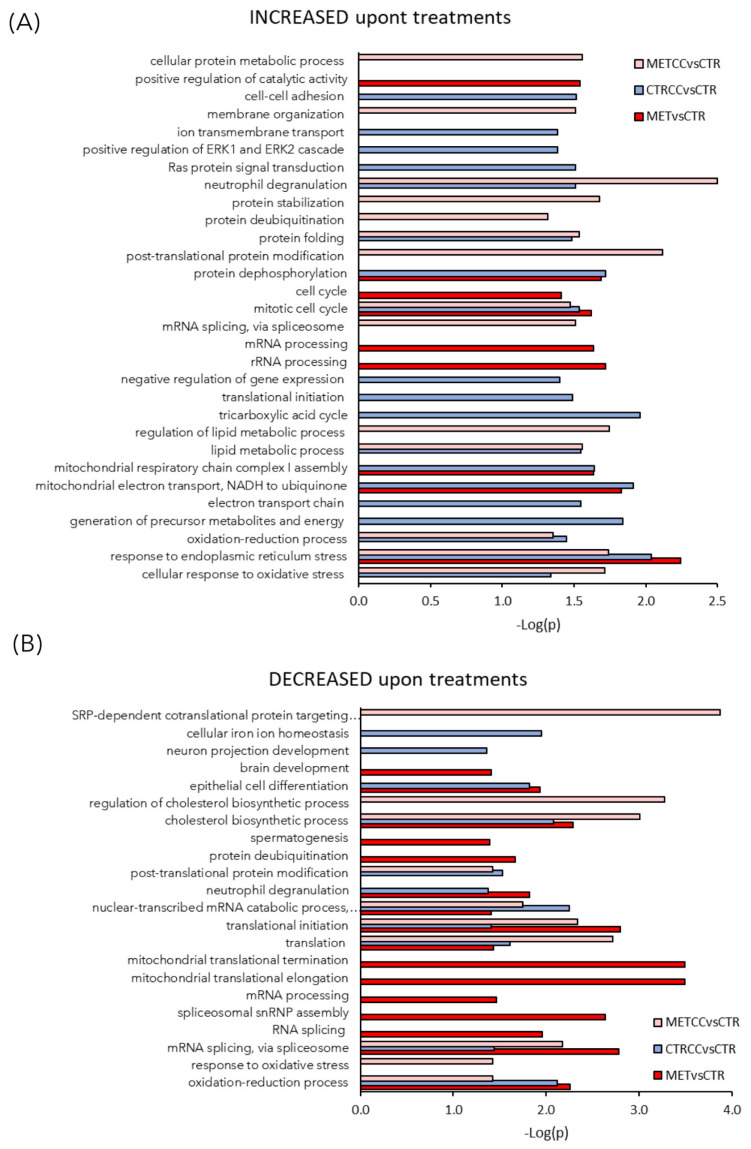
High methionine and Compound C induce proteomic changes. (A–C) Functional analysis of the proteins differentially expressed in the comparison MET versus CTR, METCC versus MET, CTRCC versus CTR. Proteins were considered differentially expressed if they were present only in one condition or showed significant *t*-test difference (Student’s *t*-test *p* value = 0.05). (**A**) GO Biological Processes increased in the three comparison (**B**) GO Biological Processes decreased in the three comparison. Functional grouping was based on Fischer’s exact test *p*-value ≤ 0.05 (i.e., −log10 ≥ 1.3) and at least three counts. In each comparison, the terms increased or decreased refer to proteins up or downregulated upon treatments.

**Figure 3:**
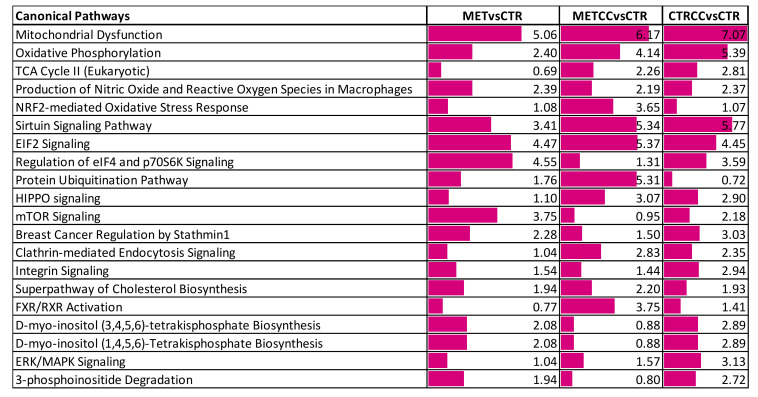
Bioinformatic analysis of the proteomic data on HepG2 cells grown for 48 h (CTR), in the presence of Compound C (CTRCC), high methionine (MET) and high methionine and Compound C (METCC). The analysis was carried out by IPA on the proteins differentially expressed among the comparison MET versus CTR, METCC versus CTR and CTRCC versus CTR. Proteins were considered differentially expressed if they were present only in one condition or showed significant *t*-test difference (Student’s *t*-test *p*-value 0.05). Functional grouping was based on Fischer’s exact test *p* value ≤ 0.05 and at least three counts. The colored bars are a visual representation of the corresponding –log *p*-value reported abreast.

These proteins were further analyzed to find possible enrichment in GO Biological Processes (Figure 2) and pathways (Figure 3), in the comparison between control and treatments with high methionine or/and Compound C. Differentially expressed proteins mainly belonged to the classes of metabolic processes, mitochondrion, and mitochondrial dysfunction, translation, RNA and protein processing and response to oxidative stress. As shown in Figure 2A,B and in Table S1, the expression of proteins related to oxidation-reduction events were altered, either up or downregulated, indicating a general impact on these processes in the presence of methionine and/or Compound C compared to the control. Many proteins involved in mitochondrial respiratory chain were upregulated by the double treatment with high methionine and Compound C. In particular, high methionine induced an increase of proteins involved in the mitochondrial electron transport and in complex I assembly, while Compound C treatment mainly increased the level of tricarboxylic acid (TCA) cycle and lipid metabolic processes (Figure 2A).

Methionine and Compound C presence upregulated proteins involved in the cellular response to oxidative stress, manly at the endoplasmic reticulum level (Figure 2A, Figure 3), while Compound C alone induced the expression of proteins involved in membrane organization, transmembrane transport, and cell–cell adhesion (Figure 2A).

Interestingly, methionine and Compound C altered sirtuin signaling pathway (Figure 3). This pathway is a master regulator of several cellular processes and known to both extend lifespan and regulate spontaneous tumor development. It is well known that S-adenosyl-methionine, sirtuin, and mTOR pathway are strictly related [35]. In keeping with these data and with results reported above, the proteomic analysis suggested that mTOR signaling was altered mainly in response to increased methionine in the medium (Figure 3).

Among the processes more altered in HepG2 cells treated with methionine and Compound C, RNA processing and mitochondrial translation were downregulated mainly by methionine, while Compound C had more impact on general translation (Figure 2B). Overall, the double treatment with high methionine and Compound C induced a decrease in the level of ribosomal proteins RPS9, RPL23A, RPL34, mitochondrial ribosomal proteins MRPL37, MRPS18C, and translational initiation factors, such as EIF1 and EIF3M, as well as proteins of the SRP (signal-recognition-particle) co-translational process (SRP14, SSR3, SRP9), suggesting a general reduction of translation in this condition (Table S4).

### 3.2. High Methionine and Compound C Induce a Metabolic Rewiring

**Figure 4:**
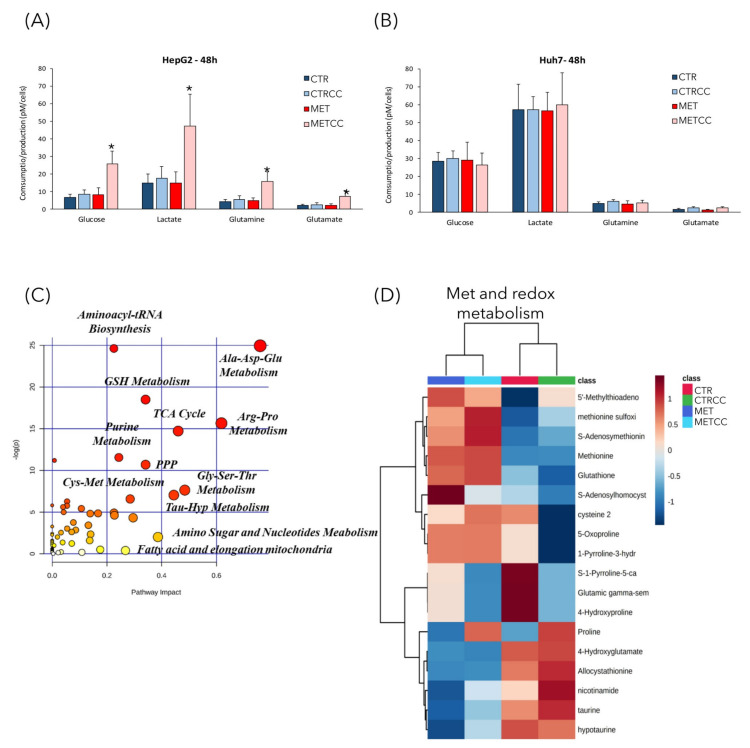

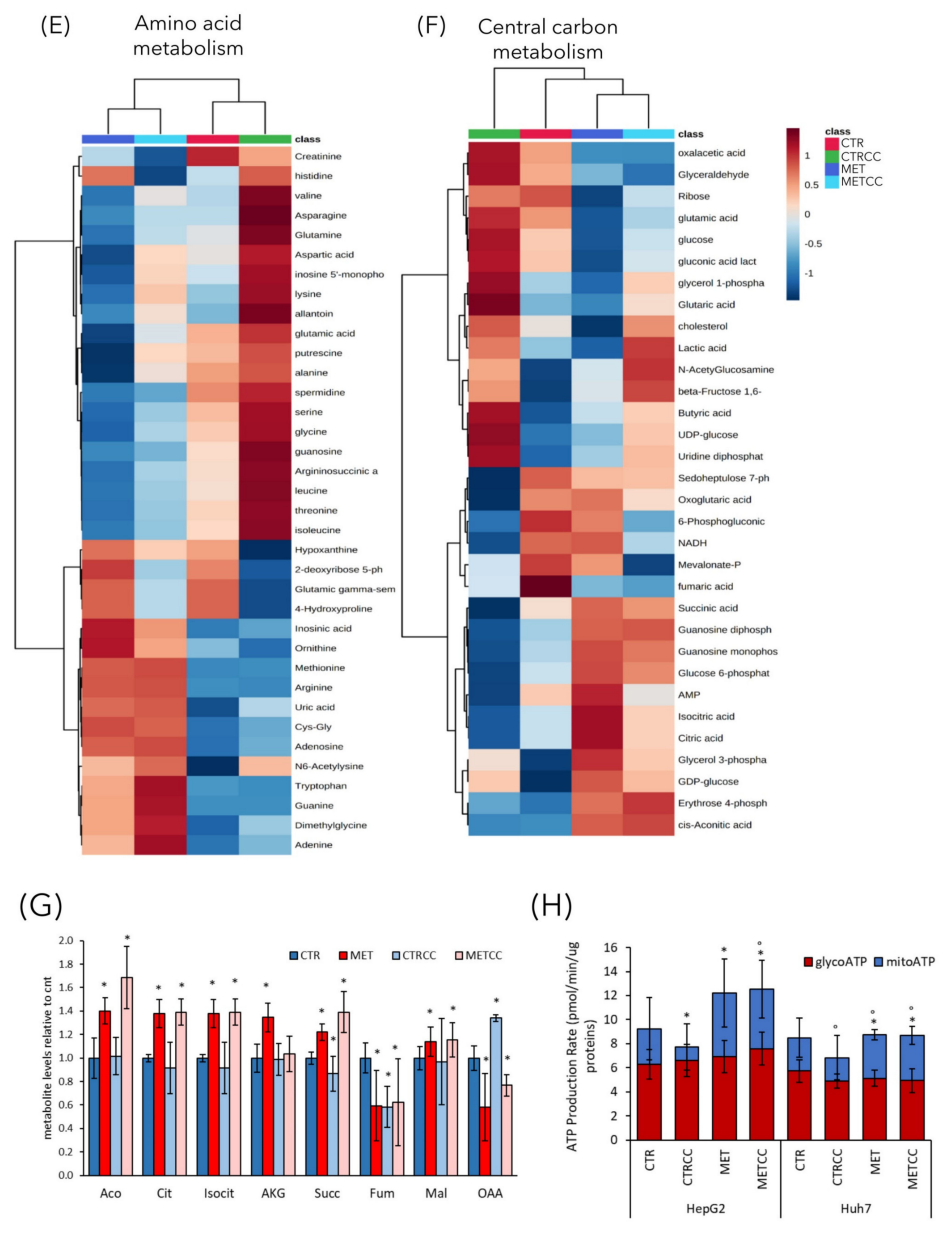
High methionine and Compound C affect cellular metabolism. (**A**,**B**) HepG2 (**A**) and Huh7 (**B**) cells were grown for 48 h in regular medium or in the presence of high methionine and/or Compound C. Media from three biological replicates were collected to measure glucose and glutamine consumption, lactate, and glutamate secretion. (**C**–**F**) HepG2 cells were grown for 48 h in regular medium (CTR), or in the presence of high methionine and/or Compound C. Metabolomics analysis was performed by GC/MS and LC/MS on five biological replicates, each analyzed in technical duplicate. (**C**) Metabolic pathways mostly affected in this analysis. The metabolic pathway analysis was performed using the MetaboAnalyst 4.0 tool. The metabolic pathways are represented as circles. Color intensity (white-to-red) and size of each circle reflects increasing statistical significance, based on the *p*-value [−log(P)] from the pathway enrichment analysis, and pathway impact value derived from the pathway topology analysis, respectively. (**D**–**F**) Hierarchical clustering heatmaps from one-way ANOVA analysis of differential metabolites belonging to (**D**) methionine and redox metabolism, (**E**) amino acid metabolism, (**F**) central carbon metabolism. The heatmaps were obtained using the MetaboAnalyst 4.0 tool. The color code scale indicates the normalized metabolite abundance. (**G**) Histogram of the level of metabolites of the TCA cycle in the four conditions in HepG2 cells, measured in the metabolomics analysis. The level of each metabolite in the control was set to 1. * *p* < 0.05 compared to control. (**H**) Seahorse adenosine triphosphate (ATP) rate assay analysis in HepG2 and Huh7 cells, grown for 48 h in regular medium or in the presence of high methionine and/or Compound C. * *p* < 0.05 for mitoATP compared to control, *p* < 0.05 for glycoATP compared to control.

Since methionine supplementation induced deep changes of proteins associated with cellular metabolism, we further investigated this feature by performing metabolomics analysis in cells grown with high methionine and/or Compound C. Extracellular metabolites were analyzed by YSI biochemical analyzer. In HepG2 cells, only the double treatment (high methionine and Compound C) showed an increase of glucose and glutamine consumption, with a proportional increase in lactate and glutamate secretion (Figure 4A). However, no changes in extracellular metabolites were observed in Huh7 cells in any condition. This difference could be due to the lower growth rate of Huh7 cells, or to their higher basal metabolism compared to HepG2 cells; indeed, their basal glucose uptake and their lactate secretion were three times higher than those of HepG2 cells (Figure 4A,B).

We then measured relative abundance of intracellular metabolites in HepG2 cells treated for 48 h with high methionine and/or Compound C, by an untargeted metabolomic GC/MS and LC/MS analysis. This analysis revealed a strong metabolic change due to high methionine, but also to Compound C addition (Supplementary Figure S1, Figure 4C). The most affected pathways were those related to amino acids metabolism, like Cys-Met (as expected upon methionine supplementation), Ala-Asp-Glu, Arg-Pro, and Gly-Ser-Thr (Figure 4C), but also those related to aminoacyl-tRNA biosynthesis, coherently with the downregulation of translation associated proteins (Figure 2B). In addition, alterations in the pathways of glutathione metabolism (which is directly associated to methionine metabolism), of the sulfur-containing molecules taurine and hypotaurine, in the pathways of purine and nucleotide metabolism and of fatty acid metabolism were identified (Figure 4C). As expected, methionine supplementation in the medium (either in the presence or absence of Compound C) increased the intracellular level of methionine, but also of its derived metabolites, such as S-adenosyl-methionine and S-adenosyl-homocysteine, which are part of the methionine cycle (Figure 4D). In addition, high methionine treatment induced an increase of cysteine, which can derive from homocysteine and serine, through conversion to cystathionine, and of glutathione, which is synthesized from Cys, Glu, and Gly. In high methionine condition there was also an increase of two direct methionine derivatives, the regulatory metabolite methylthioadenosine (MTA) and the toxic compound methionine sulfoxide (Figure 4D). Remarkably, the MTA level negatively correlates with growth potential in the liver [36,37]. In fact, it has been reported that MTA decreases after partial hepatectomy, when the replicative response of hepatocytes is higher [38], and MTA administration in vitro reduces liver cell growth [39]. Furthermore, MTA inhibits the synthesis of polyamines [36], whose level is correlated with proliferation and which were indeed decreased in high methionine condition (see spermidine and putrescine, Figure 4E). On the contrary, the MTA derivatives adenine and adenosine increased in high methionine condition (Figure 4E). High methionine also induced an increase of metabolites related to the urea cycle, such as arginine, ornithine, uric acid (Figure 4E), which was probably increased to convert excess nitrogen (given by the high methionine supplied in the medium) into urea.

### 3.3. High Methionine and Compound C Increase Mitochondrial Functionality

The metabolomics analysis revealed also that many metabolites of the TCA cycle were modulated by high methionine, with higher levels of cis-aconitate, citrate, isocitrate, α-ketoglutarate, succinate and malate and lower levels of fumarate and oxaloacetate compared to the control (Figure 4F,G).

Since the metabolomics and proteomics data suggested an increase of the TCA cycle, we measured the rate of ATP production from glycolysis and from mitochondrial respiration in HepG2 and Huh7 cells, through Agilent Seahorse technology (Figure 4H). Although the amount of ATP deriving from glycolysis was only slightly altered by treatments, cells grown in high methionine condition (with or without Compound C) produced a higher fraction of ATP through mitochondrial respiration (Figure 4H). The increase of mitochondrial-derived ATP was clearly evident in HepG2 cells and it was also confirmed, albeit with a lower increase, in Huh7 cells.

According to systems biology, the phenotype of a cell results from the interaction of several component, in which the emergent behavior is wider than the sum of their parts [40]. As a result of this, we decided to integrate the results from metabolomic and proteomic analysis, using the IPA and MetaboAnalyst softwares.

**Figure 5:**
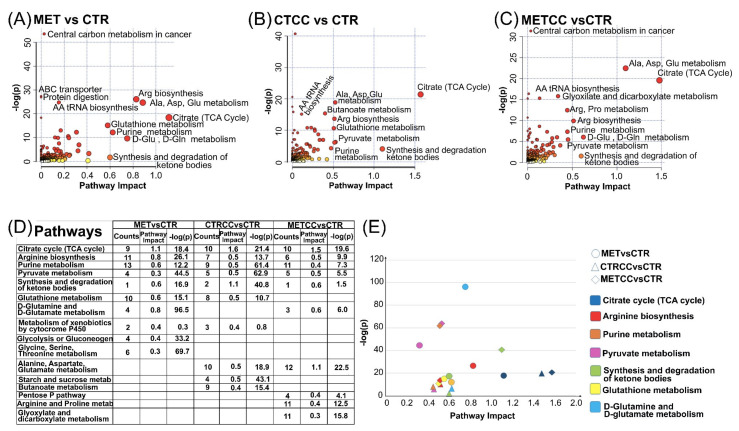
Integration of metabolomics and proteomics analysis. (**A**) Metabolic and proteomic pathways mostly affected in MET versus CTR. (**B**) Metabolic and Proteomic pathways mostly affected in CTRCC versus CTR. (**C**) Metabolic and Proteomic pathways mostly affected in METCC versus CTR. The scatter plots show a summary of the joint evidence from enrichment analysis (*p*-values) and topology analysis (pathway impact). Dots size and color (white to red) are proportional to the numbers of genes and compounds present in a pathway. (**E**) List of the 10 more significant pathways in each of the three comparisons. (**E**) Scatter plot of the common pathways among the list shown in (**D**).

This integration better highlighted that TCA cycle was one of the most affected process in all three comparisons (MET versus CTR, CTRCC versus CTR, METCC versus CTR) (Figure 5A–C). However, as reported in Figure 5D,E, focusing on the first 10 more significant pathways in terms of enrichment (*p*-values) and topology (pathway impact) in the three comparisons, the integration analysis suggested that citrate and TCA cycle were more affected by the presence of Compound C than methionine, which, in turn, had a major impact on amino acid metabolism, especially of those amino acids directly linked to the nitrogen cycle, such as Arg, Gln, and Glu. In keeping with the effect on TCA cycle, pyruvate metabolism and the synthesis and degradation of ketone bodies prevailed in the presence of Compound C if compared to the supplementation of methionine (Figure 5D,E).

Strikingly, the pentose phosphate pathway, Arg/Pro metabolism, glyoxylate, and dicarboxylate metabolism were altered only upon double treatment (Figure 5D). In addition, glutathione metabolism and metabolism of xenobiotics, which were affected by single treatments, were no more altered upon double treatment (Figure 5D). These results indicate that the combination of Compound C and high methionine does not result in a simple synergistic effect of the single treatments, but rather leads to the emergence of new features in liver cancer cell metabolism.

### 3.4. High Methionine Activates AMPK and mTOR Pathways

**Figure 6:**
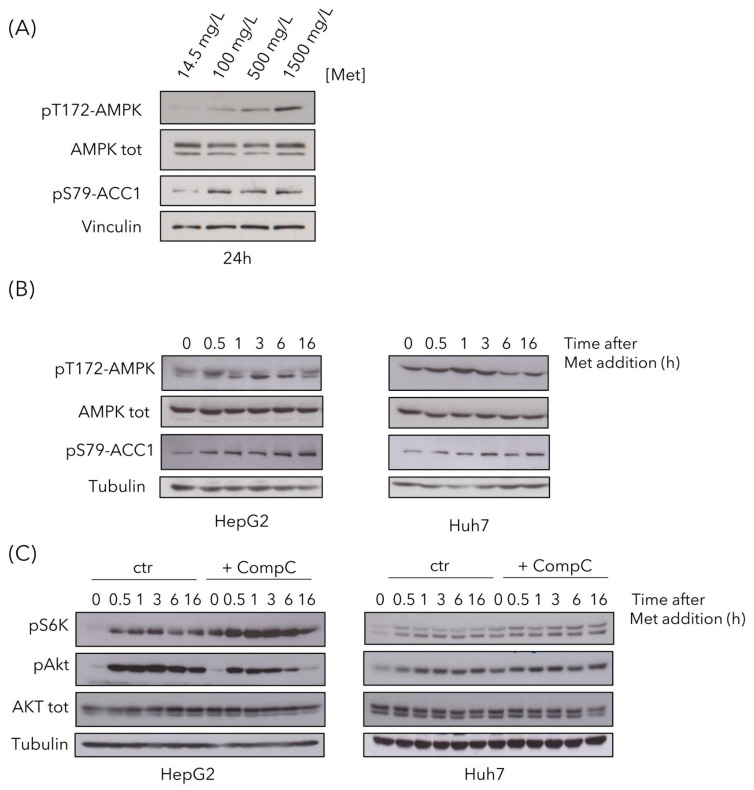
High methionine activates AMK-activated protein kinase (AMPK), mTOR and Akt pathways. (**A**) HepG2 were treated with methionine for 24 h and AMPK activation state was assayed by Western analysis, using the pT172-AMPK antibody (against pT172 in the activation loop) and using the anti-pS79-Acc1 antibody (against the target site of AMPK on Acc1). An anti-AMPK total antibody and an anti-vinculin antibody were used as controls. (**B**) HepG2 and Huh7cells were gown in control medium and 1.5 g/L methionine was added to the cultures at time 0. Samples were collected at the indicated time points to evaluate AMPK activation, using an anti-pT172-AMPK antibody and an anti-pS79-Acc1 antibody. An anti-AMPK total antibody and an anti-tubulin antibody were used as controls. (**C**) HepG2 and Huh7 cells were gown for 48 h in the absence or presence of Compound C. Then 1.5 g/L methionine was added to the cultures, and samples were collected at the indicated time points to evaluate mTOR activation, using anti-pS6K antibody and Akt activation using anti-pS473-Akt antibody. Anti-Akt total antibody and anti-tubulin antibody were used as controls.

Since we previously reported that methionine can activate Snf1/AMPK in budding yeast, we then investigated the effect of methionine supplementation in liver cancer cells. AMPK showed a dose-dependent activation after 24 h in HepG2 cells (Figure 6A), detectable as increased phosphorylation on T172, as well as increased phosphorylation of Acc1-S79, the main target of AMPK, often used as reporter of its activity. It is known that AMPK activation is mainly due to an energy reduction [41], although it can also be activated without any ATP decrease [42]. Since data presented above (Figure 4H) indicate that ATP level did not decreased, but rather increased after methionine treatment, we can speculate that AMPK activation was not a result of energy deficiency.

To better investigate AMPK activation, we performed time-course experiments in both HepG2 and Huh7 cell lines. A total of 1.5 g/L of methionine was added to cells growing in regular medium and phosphorylation of AMPK-T172 and of Acc1-S79 were detected by Western blot analysis (Figure 6B). AMPK phosphorylation, as well as the phosphorylation of its target Acc1, increased in both cell lines, with a peak at 0.5 h and 1 h after methionine addition, for HepG2 and Huh7 respectively (Figure 6B).

Since amino acids can activate mTOR, the master regulator of cell growth [43] and, since mTOR involvement was suggested by our proteomics analysis (Figure 3), we investigated the activation of the mTOR pathway. mTOR was activated in response to high methionine in the medium, as observed by increased pS6K phosphorylation (Figure 6C), in keeping with previously reported data [44], both in HepG2 and in Huh7 cells. Phosphorylation increased after 30-min treatment, remaining high until 16 h (Figure 6C). This increase was more evident in cells pre-treated with Compound C, in which the release of mTOR inhibition by AMPK resulted in a higher pS6K phosphorylation, both at time 0 and in response to high methionine (Figure 6C). High methionine also induced activation of the Akt pathway, involved in cell proliferation and survival [45], in both cell lines, although in HepG2 cells, it was less persistent over time when AMPK was inhibited (Figure 6C).

### 3.5. High Methionine Reduces Cancer Associated Phenotypes

**Figure 7:**
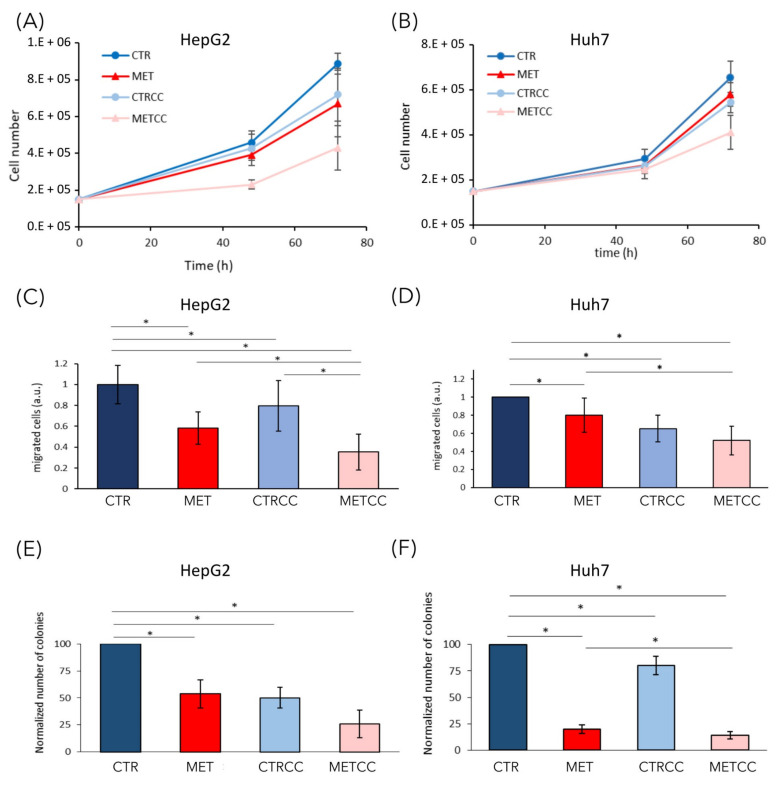
High methionine and Compound C inhibit cancer phenotypes. (**A**,**B**) Cell growth of (**A**) HepG2 and (**B**) Huh7 was monitored until 72 h in regular medium (CTR) or in the presence of 1.5 g/L methionine (MET) and/or Compound C to obtain partial AMPK inactivation (2 µM Compound C for HepG2 and 1.5 µM for Huh7). The experiments were performed at least in triplicate. (**C**,**D**) HepG2 or Huh7 were grown for 24 h in regular medium (CTR), or in the presence of high methionine and/or Compound C. Then they were starved for 24 h in the same medium without serum and migration was evaluated with a transwell assay for 24 h. (**E**,**F**) Colony formation assay of HepG2 or Huh7 grown in regular medium or in the presence of high methionine and/or Compound C. Experiments were performed in triplicate. * *p* < 0.05.

We previously showed that, in yeast cells, methionine induced a slow-down of growth rate, especially in cells lacking Snf1/AMPK activity [33]. The increase of methionine concentration in the medium (up to 1.5 g/L, versus 15 mg/L in regular medium) induced a slight slow-down of growth rate both in HepG2 and in Huh7 cell lines (Figure 7A,B). Inhibition of AMPK with Compound C slightly reduced growth rate in both cell lines, but drastically impaired growth when combined with high methionine in the medium (Figure 7A,B). These results suggest that the effect of methionine on cellular proliferation is a conserved feature that deserves further investigation.

In addition to a higher proliferation, one of the most relevant features of cancer cells is their ability to migrate and to form colonies from single cells, to give metastasis [46]. To analyze the effect of methionine on cell migration, we used Boyden chambers and migration of serum starved cells was assessed in the presence or absence of high methionine concentration and/or Compound C. Cell migration through the membrane of the transwell was significantly impaired in high methionine in both cell lines, even more when AMPK was inhibited (Figure 7C,D).

We then tested the ability of single cells to form colonies in the presence of high methionine and/or inhibition of AMPK. As shown in Figure 7E,F, colony formation was reduced by high methionine supplementation, especially in the presence of Compound C (Figure 7E,F).

Altogether, our results suggest that high methionine supplementation inhibits cancer associated phenotypes, especially in combination with AMPK inhibition.

### 3.6. The Effect of High Methionine and Compound C is Specific for Liver Cancer Cells

**Figure 8:**
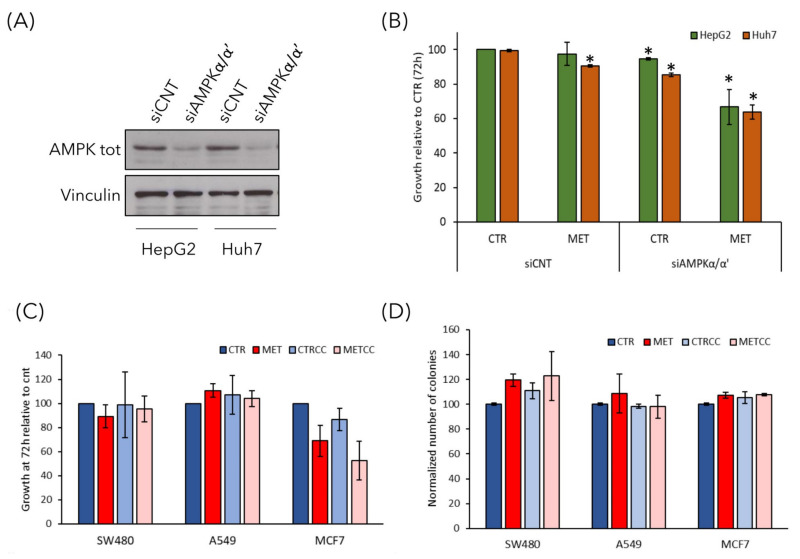
The effect of high methionine and AMPK inhibition is specific for liver cancer cells. (**A**,**B**) Gene knockdown for AMPKα/α’ in HepG2 and Huh7 cells was achieved by siRNA. (**A**) The level of endogenous AMPKα/α’ protein was detected by immunoblot by using anti-AMPK total antibody. anti-tubulin antibody was used as loading control. (**B**) Cell growth of HepG2 and Huh7 cells transfected with siCNT or siAMPKα/α’ was monitored for 72 h in regular medium (CTR) or in the presence of 1.5 g/L methionine (MET). Cell growth is expressed as a ratio on the growth in control medium. * *p* < 0.05 compared with siCNT cells. (**C**) Cell growth of SW480 colorectal cancer cells, A549 lung cancer cells, and MCF7 breast cancer cells was monitored until 72 h in regular medium (CTR) or in the presence of 1.5 g/L methionine (MET) and/or 2 µM Compound C. Cell growth is expressed as a ratio on the growth in control medium. (**D**) Colony formation assay of SW480, A549, and MCF7 cells grown in regular medium (CTR) or in the presence of high methionine and/or 2 µM Compound C.

Although Compound C has been extensively used to inhibit AMPK activity, it may also inhibit other kinases. To discern whether the effect of Compound C on growth inhibition in high methionine condition was specifically due to AMPK inhibition, we silenced the expression of AMPKα/α’ subunits in HepG2 and Huh7 cells and tested their growth in the presence of high methionine in the medium (Figure 8A,B). We found that cells with siAMPKα/α’ showed a reduced growth at 72 h in the presence of high methionine, thus, confirming that high methionine had a negative effect on growth when AMPK activity was low (either due to chemical inhibition or to reduction of the catalytic subunit). These data confirm that the effect of Compound C on growth rate was mediated by AMPK inhibition, and not by non-specific effects of the compound.

Finally, to analyze whether the effect of high methionine and AMPK inhibition was specific for liver cancer cells or could be observed also on other cancer cell types, we performed the growth assay and the clonogenic assay on three cell lines deriving from different tumors: SW480 colorectal cancer cells, A549 lung cancer cells and MCF7 breast cancer cells. High methionine and/or Compound C had no effect on the growth of SW480 and A549 cells, while high methionine slightly reduced the growth of breast MCF7 cells also in combination with AMPK inhibition (Figure 8C). However, the ability of forming colonies from single cells was not impaired in none of the cell lines tested (Figure 8D), although in MCF7 cells high methionine induced the formation of smaller colonies (not shown).

Thus, we can assume that the effect of this treatment on cancer associated phenotypes is specific for liver cancer cells.

## 4. Discussion

The role of methionine has been long investigated in many different fields [13]. Indeed, it is well known that methionine restriction extends lifespan in different model systems, from budding yeast to *Drosophila melanogaster*, *Caenorhabditis elegans*, and mammalian cells [47,48,49,50]. Methionine restriction also affects the cardiovascular system [51] and bone development [52].

On the contrary, the relationship between methionine and human cancers progression is still very ambiguous, most probably because it is cancer specific. Different studies showed that methionine restriction delays cancer progression. This was reported for instance in colon and prostate cancer animal models [53,54], as well as in breast cancer in vitro and in vivo [55,56]. On the contrary, other reports indicate that methionine supplementation induces cell-cycle arrest and transcriptional alterations in breast and prostate cancer cells [57,58].

It was reported that S-adenosyl-methionine (SAM) supplementation inhibits liver cancer cell invasion in vitro [19], by inducing changes in the methylation state of DNA, that lead to downregulation of genes involved in growth and metastasis, already known to be upregulated in liver cancer cells. In addition, in a rat model of hepatocarcinogenesis [59], as well as in a mouse model for inflammation-mediated HCC [20], SAM administration exerted a chemopreventive effect on HCC development. However, although a short-term treatment with SAM showed positive effects in the mouse model, a long-term administration did not affect tumor growth and hepatocyte proliferation [20]. Here, we explored the combination of methionine administration (the precursor of SAM) and AMPK inhibition in vitro. AMPK is a dual role kinase, being either anti- or pro-tumorigenic depending on the context, on the stage of tumor development and on the cancer type [6]. We showed that, in liver cancer cells, high methionine concentration in the medium reduces cell growth inhibits colony formation and cell migration in two different liver cancer cell lines (Figure 7) and, remarkably, these phenotypes were increased when high methionine was combined with AMPK inhibition (Figure 7 and Figure 8). This is perfectly in line with what we observed in budding yeast, where growth rate reduction due to methionine in the medium was largely dependent on the presence of an active Snf1/AMPK pathway [33]. Moreover, methionine induces an activation of Snf1/AMPK in *S. cerevisiae*, as we observe in liver cancer cells (Figure 6), highlighting that AMPK involvement in the response to methionine is a conserved feature in eukaryotic systems. An intriguing aspect of methionine response is the activation of the mTOR pathway (Figure 6), which is coherent with the reported effect of SAM (the first metabolite of methionine) on mTOR activation through SAM-sensor upstream of mTOR1 (SAMTOR) [44]. However, the intracellular level of most amino acids was downregulated in cells grown in high methionine (Figure 4), and this probably leads to the observed downregulation of proteins involved in translation and tRNA synthesis (Figure 1, Figure 2, Figure 4 and Figure 5) and to the reduction of growth rate (Figure 7). This condition—active mTOR with reduced translational capacity—reminds the condition of cycloheximide treatment, in which mTOR phosphorylates pS6K [60] although growth is impaired, thus, producing the effect of a counter circuit in the cell.

High methionine has also a strong effect on metabolome and proteome remodeling, as also reported in yeast cells [33], especially when combined with Compound C. In fact, reduction of intracellular amino acid levels and alterations in metabolites of the TCA cycle were found in both yeast and liver cancer cells. Interestingly, the observed increase of proteins and metabolites of the TCA cycle could be a direct consequence of methionine metabolism to homocysteine, which can be then converted to α-keto-butyrate and enter the mitochondria. However, in yeast cells, the effect of methionine on mitochondrial functionality was much more evident, probably due to the fact that yeast cells have a fermentative metabolism in the presence of glucose. On the contrary, in human cells, which have a mixed respirative and fermentative metabolism, the effect on mitochondria better emerges by integrating metabolomics and proteomics data. These results, together with the reduction of polyamine levels (which are associated to growth rate), could explain at least in part the observed reduction of cancer phenotypes.

Why does methionine metabolism have this anti-tumor role on liver cancer cell lines, contrary to other cancer cells? It should be noted that methionine metabolism in the liver is very peculiar, since the liver is the organ where most of the methionine is converted to S-adenosyl-methionine and where only the gene MAT1A is expressed. Therefore, most of the observed effects could be due to this liver-specific metabolism, although we cannot exclude the possibility that methionine could carry out also other functions more related to protein synthesis. In fact, Mato and Lu suggest that liver cancer cells, in contrast to normal non-proliferating, differentiated hepatocytes, tend to utilize methionine mainly for protein synthesis [17].

An interesting translational application of our results could be to directly increase methionine uptake through the diet, both in animal models and in human patients. Methionine should easily reach the liver, since it is the physiological organ where it is metabolized. It should have no side effect on normal hepatocytes, since SAM was shown to have negligible effects on primary untransformed liver cells [19]. Therefore, further investigations should explore the possibility that alterations in methionine dietary consumption, in combination with pharmacological treatments, could have clinically relevant outcomes in liver cancer patients.

## Supplementary Materials

The following are available online at https://www.mdpi.com/2073-4409/9/11/2491/s1, Table S1:Summary of the proteomic results comparing MET versus CTR, METCC versus MET, CTRCC versus CTR; Table S2: Proteins differentially expressed the comparison MET versus CTR; Table S3: Proteins differentially expressed the comparison CTRCC versus CTR; Table S4: Proteins differentially expressed the comparison METCC versus CTR; Figure S1: Hierarchical clustering heatmap from One-way ANOVA analysis of the entire set of metabolites differentially expressed.
