# Supplementary material for: Methionine Supplementation Affects Metabolism and Reduces Tumor Aggressiveness in Liver Cancer Cells
Source: Cells. 2020 Nov 16;9(11):2491. doi: 10.3390/cells9112491 (PMC7696226; doi:10.3390/cells9112491)

SUPPLEMENTAL INFORMATION

Supplementary Tables

Table S1. Summary of the proteomic results comparing MET versus CTR, METCC versus MET, CTRCC versus CTR. Proteomic data were analysed by Perseus software (version 1.5.5.3, www.biochem.mpg.de/mann/tools). Proteins were considered differentially expressed if they were present only in one condition or showed significant t-test difference (Student's T-test *p* value ≤ 0.05). In each comparison the terms increased or decreased refer to proteins up- or down-regulated in CTR, respectively.

Table S2. Proteins differentially expressed the comparison MET vs CTR. Proteins were considered differentially expressed if they were present only in one condition or showed statistically significant difference (Student's T-test *p* value ≤ 0.05).

Table S3. Proteins differentially expressed the comparison CTRCC vs CTR. Proteins were considered differentially expressed if they were present only in one condition or showed statistically significant difference (Student's T-test *p* value ≤ 0.05).

Table S4. Proteins differentially expressed the comparison METCC vs CTR. Proteins were considered differentially expressed if they were present only in one condition or showed statistically significant difference (Student's T-test *p* value ≤ 0.05).

Supplementary Figure S1 (related to Fig. 3) Hierarchical clustering heatmap from One-way ANOVA analysis of the entire set of metabolites differentially expressed.

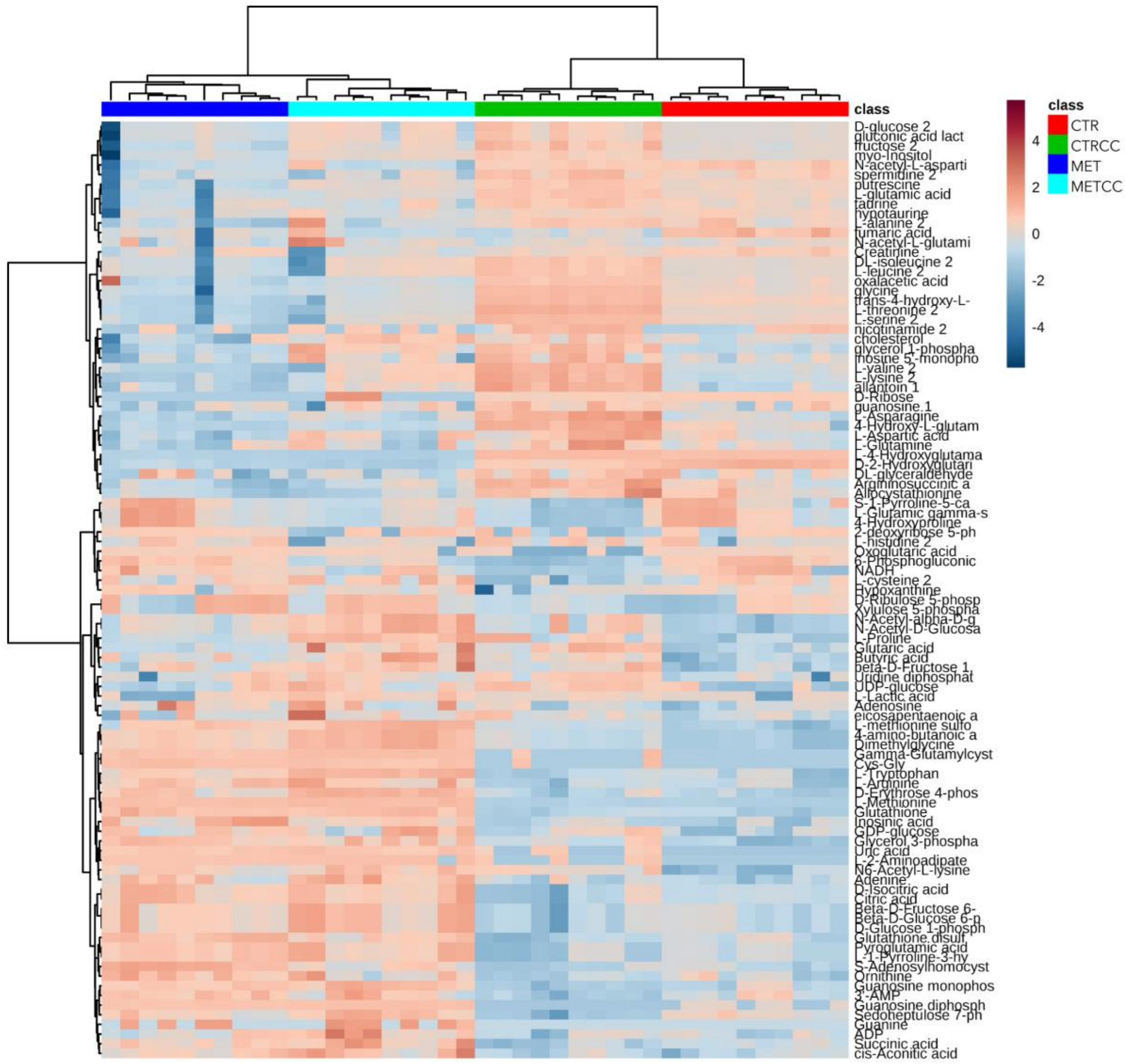

Supplement: Supplementary file 1 [file cells-09-02491-s001.zip › cells-898743-supplementary/cells-898743 Supplementary infomation (table + fig).pdf]
